# Chromodomain helicase/ATPase DNA binding protein 1-like protein expression predicts poor prognosis in nasopharyngeal carcinoma

**DOI:** 10.3892/etm.2014.2017

**Published:** 2014-10-13

**Authors:** FA-REN SU, JING-HUA DING, LIN BO, XIN-GANG LIU

**Affiliations:** Department of Otorhinolaryngology and Head and Neck Surgery, Shandong People’s Armed Police Corps Hospital, Jinan, Shandong 250014, P.R. China

**Keywords:** nasopharyngeal carcinoma, CHD1L, prognosis, biomarker

## Abstract

Nasopharyngeal carcinoma (NPC) is a malignancy with a high metastatic ability. Recent studies have implicated the role of chromodomain helicase/ATPase DNA binding protein 1-like (CHD1L) gene as a novel oncogene; however, the functional role of CHD1L in NPC remains unknown. The aim of this study was to evaluate the clinical significance of CHD1L positivity in NPC. CHD1L protein expression was examined by performing western blot analysis of 30 fresh NPC tissues and conducting immunohistochemistry tests of 133 NPC samples between December 1, 2005 and December 1, 2009. The correlations of CHD1L expression status with clinicopathological features and prognosis were investigated. Immunohistochemical analysis showed that 88 of 133 (66.2%) paraffin-embedded NPC biopsies exhibited positive expression of CHD1L, but all non-cancerous nasopharyngeal specimens were negative for CHD1L expression. In addition, positive CHD1L expression was strongly associated with an advanced clinical stage (P=0.016), recurrence (P=0.002) and the metastasis status (P=0.031) of NPC. Kaplan-Meier survival analysis demonstrated that patients with CHD1L-positive NPC had significantly shorter overall survival (P<0.001). Furthermore, the multivariate analysis indicated that CHD1L protein expression was an independent prognostic factor for overall survival (hazard ratio, 7.916; 95% confidence interval, 2.067–16.034; P=0.003) in patients with NPC. These results indicate that CHD1L is a prognostic marker for NPC.

## Introduction

Nasopharyngeal carcinoma (NPC) is derived from the epithelial cells that cover the surface of and line the nasopharynx (1). Worldwide, NPC is most prevalent in China, with an age-standardized incidence rate of >20 per 100,000 individuals (2). NPC, often accompanied by early lymph node metastasis, is hard to detect due to the hidden location and lack of clinical manifestations at the early stages (3). The present clinical screening method for NPC is mainly dependent on detecting Epstein-Barr virus (EBV) infection in the serum, but not all NPC carcinogenesis is associated with EBV; furthermore, EBV infection can be observed in a number of other diseases (4). The use of EBV-related detection alone for NPC screening cannot meet the requirements as a diagnostic marker; therefore, finding the molecular biomarkers for NPC is necessary to improve the treatment regimes and predict the prognosis for patients with NPC.

Amplification of 1q21 is a frequent genetic alteration in a number of solid tumors (5). In the last decade, a novel oncogene, chromodomain helicase/ATPase DNA binding protein 1-like (CHD1L) gene, has been isolated from the 1q21 amplicon. CHD1L belongs to the SNF2 ATPase superfamily with a carboxy-terminal macrodomain, which is involved in DNA damage repair and cell cycle progression (6,7). In a previous study amplification of CHD1L at the protein level was detected in most hepatocellular carcinomas (HCCs); and CHD1L-transfected cells were shown to possess a strong oncogenic ability (8). Furthermore, studies have shown that CHD1L expression was significantly associated with venous infiltration, microsatellite tumor nodule formation, an advanced tumor stage and poor survival in HCC (9,10). Although CHD1L is implicated in the pathogenesis of several types of cancer, the expression of CHD1L and its significance in NPC have not been well documented. In this study, the expression of CHD1L was investigated to evaluate the prognostic role in patients with NPC with long-term follow-up.

## Materials and methods

### Patient samples

NPC biopsies were collected from 30 fresh samples as well as 133 primary NPC and 133 paired adjacent nasopharyngeal tissues at the Department of Otorhinolaryngology and Head and Neck Surgery, Shandong People’s Armed Police Corps Hospital (Jinan, China) between December 1, 2005 and December 1, 2009. No patients received treatment prior to surgery. The cause of mortality was determined according to medical records.

Fresh and formalin-fixed paraffin-embedded tissues were collected from the hospital at the time surgical resections were performed. All biopsies were histologically confirmed by two pathologists in a blind manner. The clinical stage of NPC was determined according to the tumor, node and metastasis (TNM) classification system of the American Joint Committee on Cancer/Union for International Cancer Control, and the histological type was designated according to the World Health Organization (WHO) criteria (11). Follow-up data included survival status and disease status (disease-free, recurrence or metastasis), along with dates of the events and cause of mortality.

All the patients provided informed consent prior to this study. This study was approved by the Ethics Committee of Shandong People’s Armed Police Corps Hospital and performed in line with the Declaration of Helsinki.

### Western blot analysis

Frozen NPC or non-tumor tissue samples were homogenized in radioimmunoprecipitation assay buffer (Qiagen, Shanghai, China). Following centrifugation at 15000 × g, 4°C for 20 min, 70 μg protein samples were run on a 12.5% SDS-PAGE gel and transferred to polyvinylidene difluoride membranes (Millipore, St. Charles, MO, USA). Subsequent to blocking non-specific binding sites for 60 min with 5% fat milk, the membranes were incubated with rabbit monoclonal antibodies against CHD1L (1:1,000; Millipore) and GAPDH (1:1,000; Millipore) at 4°C overnight, respectively. The membranes were then washed with Tris buffered saline with Tween 20 (TBST) three times, for 15 min each time, and incubated with horseradish peroxidase-conjugated anti-rabbit secondary antibodies (1:10,000; Millipore) for 60 min at room temperature. The membrane was developed by an enhanced chemiluminescence system (Millipore) following washing with TBST three times. The intensity of the protein bands was determined by densitometry using Image J software (National Institutes of Health, Bethesda, MD, USA).

### Histological assessment

Paraffin-embedded sections of NPC samples were analyzed for the localization of CHD1L protein using anti-CHD1L antibody (1:50; Millipore) as described previously (10). The absence of primary antibody served as a negative control. The degree of immunohistochemical staining was evaluated independently by two pathologists blinded to the study, and a consensus was reached. CHD1L nuclear immunoreactivity was scored using a semiquantitative scoring system as follows: 0, no staining; 1, weak staining; 2, moderate staining; 3, strong staining. For statistical analysis, 0 and 1 were classified as CHD1L-negative; 2 and 3 were classified as CHD1L-positive.

### Statistical analysis

Statistical analyses were processed with SPSS version 17.0 (SPSS, Inc., Chicago, IL, USA). The χ^2^ test was performed for categorical data. The Kaplan-Meier method and log-rank test were used for survival analyses. Univariate and multivariate Cox regression models were used to assess the correlations between CHD1L status and the risk of mortality. P<0.05 was considered to denote a statistically significant difference.

## Results

### Upregulation of CHD1L in human NPC

**Figure 1:**
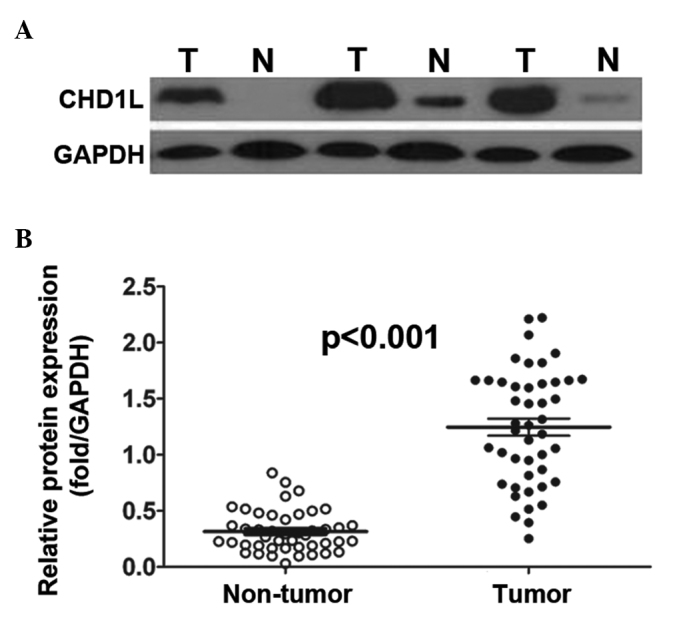
Increased CHD1L protein expression in NPC tissues as shown by western blotting. (A) Representative result of CHD1L protein expression in three paired NPC and matched adjacent non-tumor tissues. (B) Relative CHD1L protein expression levels in NPC and non-tumor tissues (CHD1L/GAPDH, n=30, P<0.001). Bars represent the mean ± standard deviation. CHD1L, chromodomain helicase/ATPase DNA binding protein 1-like; NPC, nasopharyngeal carcinoma; T, tumor; N, non-tumor.

The expression of CHD1L protein was detected by western blotting in the 30 paired NPC cancerous tissues and their corresponding adjacent non-cancerous tissues. Western blot analyses showed that CHD1L expression was markedly elevated in NPC cancerous tissues in comparison with their corresponding non-cancerous tissues (P<0.001, Fig. 1).

**Figure 2:**
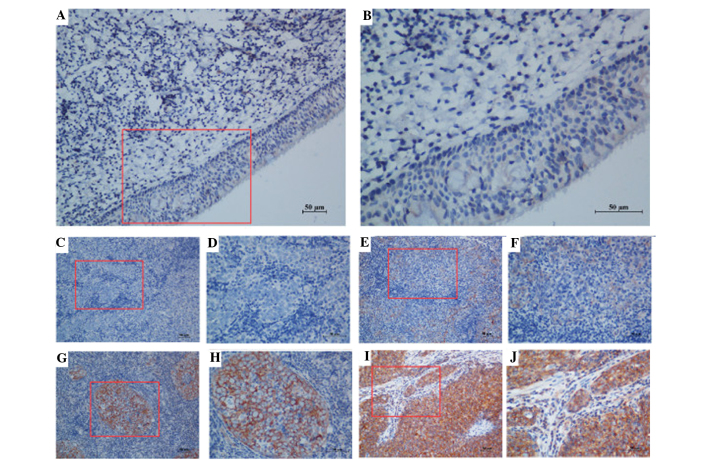
Immunohistochemical staining of CHD1L in NPC tissues. (A and B) CHD1L-negative staining in normal nasopharyngeal epithelium tissue (negative control): (A) magnification, ×200; (B) magnification, ×400. (C and D) CHD1L-negative staining in NPC tissue: (C) magnification, ×200; (D) magnification, ×400. (E and F) Weak staining of CHD1L in the cytoplasm: (E) magnification, ×200; (F) magnification, ×400. (G and H) Moderate staining of CHD1L in the cytoplasm: (G) magnification, ×200; (H) magnification, ×400. (I and J) Strong staining of CHD1L in the cytoplasm (I) magnification, ×200; (J) magnification, ×400. CHD1L, chromodomain helicase/ATPase DNA binding protein 1-like; NPC, nasopharyngeal carcinoma.

Following the western blotting, CHD1L expression in 133 NPC specimens was evaluated by immunohistochemistry. Among the 133 NPC biopsies, 66.2% (88/133) of the NPC samples were classified as positive for CHD1L expression. Representative images are shown in Fig. 2. Immunohistochemical staining of CHD1L was predominantly located in the cytoplasm. By contrast, the 133 adjacent tissue specimens were negative for CHD1L expression. These data suggest that CHD1L protein was highly expressed in NPC tissues.

### Association of CHD1L protein expression with the clinicopathological characteristics of human NPC

Table I summarizes the association of CHD1L protein expression, detected by immunohistochemical staining, with clinicopathological parameters in 133 patients with NPC. High CHD1L expression was closely associated with an advanced clinical stage of NPC (P=0.016). In addition, a significant difference was observed in CHD1L expression in patients categorized according to recurrence status (P=0.002). The expression levels of CHD1L protein in patients with NPC with positive recurrence were significantly higher than those in patients without recurrence. Positive metastasis also correlated with higher CHD1L expression (P=0.031). No significant association was observed between CHD1L expression and age, gender, T-stage, N-stage or WHO histological type (Table I).

### Association of CHD1L protein expression with the prognosis of human NPC

**Figure 3:**
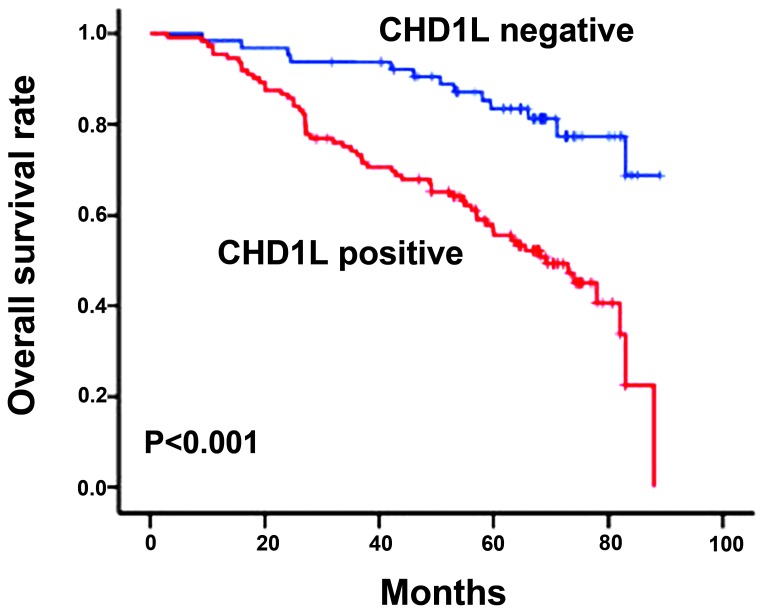
Kaplan-Meier curves for overall survival according to CHD1L status in 133 patients with NPC stratified by CHD1L immunoreactivity (negative versus positive). CHD1L positivity possesses an unfavorable prognostic value in NPC, as patients with CHD1L-positive nasopharyngeal tumors have a significantly shorter overall survival time compared with patients with NPC with CHD1L-negative tumors. CHD1L, chromodomain helicase/ATPase DNA binding protein 1-like; NPC, nasopharyngeal carcinoma.

The association of CHD1L protein expression with the prognosis of human NPC was also evaluated. The log-rank test and Kaplan-Meier analysis were used to determine the effect of classic clinicopathological characteristics, including age, gender, clinical stage, T-stage, N-stage, WHO histological type, recurrence and metastasis, and CHD1L expression on survival. The log-rank test showed that the low CHD1L expression group had a significantly improved survival time, whereas the high CHD1L expression group had a shorter survival time (P<0.001, Fig. 3). In addition, clinical stage, recurrence and metastasis showed strong correlations with survival in Kaplan-Meier analysis and log-rank tests (for clinical stage, P=0.002; for recurrence and metastasis, P<0.001; Table II).

Multivariate survival analysis, which included CHD1L expression level, clinical stage, recurrence and metastasis, was used to determine whether CHD1L expression level was an independent prognostic factor. In this analysis, as well as clinical stage, recurrence and metastasis, CHD1L expression (hazard ratio, 7.916; 95% confidence level, 2.067–16.034; P=0.003) was an independent prognostic factor for patients with NPC (Table III).

## Discussion

At present, the prognosis of NPC remains unsatisfactory, and NPC represents an invasive, rapidly proliferating tumor; therefore, it is necessary to identify prognostic biomarkers that are independently correlated with tumor prognosis and aggressiveness. In the present study, the CHD1L expression status was examined by immunohistochemistry in 133 patients with NPC in relation to survival as well as clinical and pathological features. The data showed that CHD1L was upregulated in NPC and found in 66.2% of cases. Significant correlations were also observed between CHD1L positivity and poor clinical outcome, independent of other characteristics. The results indicate that CHD1L positivity may be considered as a good prognostic marker for NPC.

According to a recent study, CHD1L can be considered to be a novel independent biomarker for progression, prognosis and survival in several types of solid tumor (12). This accumulated knowledge about the functions of CHD1L could facilitate a search for targeted treatments in specific subtypes of tumors. Data have shown that positive expression of the CHD1L protein is significantly associated with the metastasis of ovarian carcinoma; therefore, CHD1L protein expression, as examined by immunohistochemistry, may act as a novel prognostic biomarker for patients with ovarian carcinoma (13). In mice, CHD1L activates the expression of Sparc/osteonectin, cwcv and kazal-like domains proteoglycan 1 (SPOCK1), which activates Akt signaling to block apoptosis and invasion by HCC cells, and levels of SPOCK1 increase with the progression of human HCC (14). Furthermore, overexpression of CHD1L in patients with colorectal carcinoma has been shown to correlate with a large tumor size, deep tumor invasion, a high histological grade and poor disease-free survival (15). CHD1L-transfected cells have been observed to exhibit a strong oncogenic ability, increasing the tumorigenicity in nude mice, which could be effectively suppressed by small interfering RNA targeting CHD1L. In addition, a functional study by Ji *et al* (15) showed that overexpression of CHD1L could promote G_1_/S-phase cells and inhibit apoptosis. It was recently demonstrated that, in bladder cancer, CHD1L overexpression was significantly correlated with the histological grade and stage of the tumor (16). Multivariate analysis further demonstrated that CHD1L was an independent prognostic factor for patients with bladder cancer (16). These data suggest that CHD1L may play an important role in promoting tumorigenesis or progression; however, to date, the expression and clinical significance of CHD1L in NPC have not been explored. In this present study, therefore, the aim was to analyze CHD1L protein expression in tumor tissue and to assess its prognostic significance for NPC.

In the present study, CHD1L expression in NPC specimens was investigated using western blot analysis. The results showed that the CHD1L protein levels were significantly increased in tumor tissue samples compared with those in the adjacent non-tumor tissue samples. Furthermore, immunohistochemical analysis demonstrated high CHD1L expression in 66.2% of cases of NPC. It was additionally found that a high expression of CHD1L correlated significantly with an advanced clinical stage, recurrence and the metastasis status of NPC, indicating that an increase in CHD1L expression could promote tumor growth and invasion. These results suggest that CHD1L may play an important role in the tumorigenesis or progression of NPC.

In the Kaplan-Meier survival analysis, patients with positive CHD1L expression had a significantly shorter overall survival time than patients who were negative for CHD1L expression. The univariate analysis showed that increased expression of CHD1L in NPC tissues correlated significantly with overall survival. Cox hazard ratio regression analyses further demonstrated that CHD1L expression was an independent prognostic factor in patients with NPC. These results indicate that CHD1L could serve as a valuable prognostic biomarker for patients with NPC.

Overall, it has been demonstrated that CHD1L is an independent prognostic factor for survival in patients with NPC and its positive expression could be a potential prognostic biomarker for clinical application in these patients. Prospective clinical studies of CHD1L as a novel biomarker in NPC are warranted.

## Figures and Tables

**Table I:** Association of CHD1L expression with clinicopathological parameters in 133 patients with nasopharyngeal carcinoma.

		CHD1L expression (n)		
			
Parameters	N	Positive	Negative	P-value
Age (years)				
<48	50	31	19	NS
≥48	83	57	26	
Gender				
Male	83	53	30	NS
Female	50	35	15	
Clinical stage				
I–II	34	15	19	0.016
III–IV	99	73	26	
T-stage				
T1–T2	45	28	17	NS
T3–T4	88	60	28	
N-stage				
N0	39	25	14	NS
N1–N3	94	63	31	
WHO histology				
II	39	26	13	NS
III	94	62	32	
Recurrence				
No	98	58	40	0.002
Yes	35	30	5	
Metastasis				
No	100	61	39	0.031
Yes	33	27	6	

CHD1L, chromodomain helicase/ATPase DNA binding protein 1-like; NS, not significant; WHO, World Health Organization.

**Table II:** Univariate analysis of different prognostic variables in 133 patients with nasopharyngeal carcinoma.

Variables	Subset	HR	95% CI	P-value
Patient gender	Male vs*.* female	1.987	0.608–4.092	NS
Patient age	<48 vs*.* ≥48	1.566	0.465–3.853	NS
Clinical stage	I–II vs*.* III–IV	6.814	1.031–14.613	0.002
T-stage	T1–T2 vs*.* T3–T4	2.380	0.738–5.046	NS
N-stage	N0 vs*.* N1–N3	1.778	0.732–4.028	NS
WHO histological type	II vs*.* III	1.458	0.689–3.076	NS
Recurrence	No vs*.* yes	8.916	1.021–19.656	<0.001
Metastasis	No vs*.* yes	8.421	1.042–17.975	<0.001
CHD1L	Negative vs*.* positive	10.204	1.833–30.225	<0.001

CHD1L, chromodomain helicase/ATPase DNA binding protein 1-like; HR, hazard ratio; CI, confidence interval; NS, not significant; WHO, World Health Organization.

**Table III:** Multivariate analysis of different prognostic variables in 133 patients with nasopharyngeal carcinoma.

Variables	Subset	HR	95% CI	P-value
Clinical stage	I–II vs*.* III–IV	6.193	1.011–13.392	0.006
Recurrence	No vs*.* yes	6.928	0.922–13.556	0.005
Metastasis	No vs*.* yes	6.893	1.023–13.528	0.005
CHD1L	Negative vs*.* positive	7.916	2.067–16.034	0.003

CHD1L, chromodomain helicase/ATPase DNA binding protein 1-like; HR, hazard ratio; CI, confidence interval; NS, not significant; WHO, World Health Organization.
